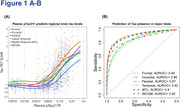# Prediction of regional brain tau levels in early Alzheimer’s disease using plasma pTau217

**DOI:** 10.1002/alz.095793

**Published:** 2025-01-09

**Authors:** Viswanath Devanarayan, Arnaud Charil, Thomas Doherty, Daniel A. Llano, Yuanqing Ye, Erica Andreozzi, Jin Zhou, Pallavi Sachdev, Larisa Reyderman, Harald Hampel, Lynn D Kramer, Shobha Dhadda, Michael C. Irizarry

**Affiliations:** ^1^ Eisai Inc., Nutley, NJ USA; ^2^ Eisai Inc., Hatfield, hertfordshire United Kingdom; ^3^ University of Illinois, Urbana Champagne, IL USA

## Abstract

**Background:**

PET quantification of brain tau pathology aids in Alzheimer’s disease staging and patient screening. This study assesses whether the phosphorylated to nonphosphorylated plasma Tau217 ratio (pTau217R) predicts regional tau PET standardized uptake value ratio (SUVR) and accurately identifies subjects with different levels of tau accumulation.

**Method:**

Plasma pTau217 and non‐phosphorylated tau217 concentrations were quantified via immunoprecipitation‐mass spectrometry. Predictive models for MK6240 tau PET SUVR were developed and validated using a 60‐40 random split of a clinical trial cohort comprising 242 amyloid‐β positive early Alzheimer’s disease individuals. The stochastic gradient boosting algorithm was employed to construct models for concurrently predicting SUVR values across various brain regions. Additional analyses explored whether integrating additional predictors (e.g., cognitive assessments, fluid biomarkers, structural MRI, and amyloid PET) into the model could improve the prediction performance of pTau217R. Model performance was cross‐validated within the training set and evaluated further in a hold‐out test set.

**Result:**

pTau217R‐based models accurately predicted tau PET uptake across various brain regions, with R2 values ranging from 0.49 to 0.65. The maximum SUVR values reliably predicted across these brain regions fell within the range of 1.87 to 2.3. The area under the receiver operating characteristic curve for detecting tau presence ranged from 84% to 95% across the six Braak stages and cortical regions, maintaining consistent performance at higher tau accumulation levels (Figure 1A‐B; cortical regions). Integrating additional predictors did not improve pTau217R’s performance. Therefore, using pTau217R alone in predicting tau PET SUVR reduced the need for tau PET scans by up to 65%, particularly in identifying low tau concentrations within cortical grey matter, while maintaining a 5% false negative rate.

**Conclusion:**

Our study demonstrates the robust predictive ability of pTau217R in estimating regional brain tau levels among Aβ+ early Alzheimer’s disease patients, accurately discerning individuals with varying degrees of tau accumulation while notably reducing the need for tau PET scans. The simultaneous prediction of continuous SUVR values across multiple brain regions enables pathological disease staging, enhances practicality, and maximizes accessibility for patients, thereby providing greater flexibility in patient screening and monitoring procedures for both clinical trials and real‐world clinical settings.